# Transcriptional regulation mechanism of flavonoids biosynthesis gene during fruit development in *astragalus membranaceus*

**DOI:** 10.3389/fgene.2022.972990

**Published:** 2022-09-06

**Authors:** Pengfei Hu, Suriguga, Ming Zhao, Shaoqing Chen, Xiaohua Wu, Quan Wan

**Affiliations:** Affiliated Hospital of Inner Mongolia Minzu University, Inner Mongolia Minzu University, Tongliao, China

**Keywords:** A. *membranaceus*, flavonoid, development, gene expression, regulation mechanism

## Abstract

*Astragalus membranaceus*, as an important medicinal plant, are an excellent source of flavonoids. Flavonoid compounds in *A. membranaceus* have been widely used in medicine and supplement, but known of the molecular mechanism of flavonoid biosynthesis is still very few. Here, we analyzed the association between flavonoid content and gene expression pattern during six different fruit developmental stages. Sixteen gene expression trends were significantly identified, involving 8,218 genes. The gene expression trend in profile 0 was positively correlated with flavonoid content, while the gene expression trend in profile 79 was negatively correlated with flavonoid content at six developmental stages. The expression level of genes involved in the general phenylpropane pathway was higher than that of genes involved in the flavonoid biosynthesis pathway. A total of 37 genes involved in flavonoid synthesis were identified in *A. membranaceus*. The expression pattern of flavonoid-related genes was highly correlated with flavonoid content. Our study deepened the understanding of the flavonoid synthesis mechanism and provided useful resources for future studies on the high flavonoid molecular breeding of *A. membranaceus*.

## Introduction

*Astragalus membranaceus*, belonging to Fabaceae, is a traditional medicinal plant in China, with strong medicinal qualities. *A. membranaceus* mainly consists of two varieties, *A. membranaceus* (Fisch.) Bunge var. Mongholicus (Bunge) P.K. Hsiao and *A. membranaceus* (Fisch.) Bunge var. Membranaceus (Wang et al., 2016; Liu et al., 2017). *A. membranaceus* is the main source of Radix Astragali, which is a well-known and widely used traditional Chinese medicine (Chen et al., 2020). For thousands of years, Radix Astragali has been used in traditional Chinese medicine to treat weakness of the spleen and the stomach, languid and night sweats (Li et al., 2014; Chen et al., 2020). In addition, Radix Astragali is used to treat clinically treat respiratory, cardiovascular, immunological, and hepatic diseases (Shan et al., 2019; Chen et al., 2020; Zhang et al., 2020; Su et al., 2021). Radix Astragali is mainly used to produce flavonoids, amino acids, polysaccharides, triterpenoid saponins and various trace elements (Man-Xiu, 2009; Fu et al., 2014; Li et al., 2014). Radix Astragali contain more than 63 flavonoids, including isoflavonones, isoflavans, pterocarpans, flavonones and chalcones (Lin et al., 2000; Lukacin et al., 2003; Song et al., 2007; Bratkov et al., 2016).

Flavonoids are a large group of secondary metabolites widely distributed in plants, including vegetables, fruits and other food crops (Shen et al., 2022). Flavonoids are ubiquitous in plant tissues, mainly in leaves, flowers and seeds (Shen et al., 2022). Flavonoids, including flavonols, flavanols and anthocyanins, are found in the leaf of *Ginkgo biloba* (Okhti et al., 2021). Meanwhile, flavonoid glycosides and terpenes are extracted from the xylem of *G. biloba*, which are secondary metabolites of antibacterial and antioxidant activity (Wang L. et al., 2020). The main flavonoid compounds of *Fagopyrum tataricum* malt are rutin, orientin, quercetin-3-O-robinobioside, isovitexin, vitexin and isoorientin, of which rutin accounts for 0.8–1.7% of the dry weight of *F. tataricum* (Mansur et al., 2020). 147 flavonoids are found in *F. tataricum*, and flavonoid compounds are significantly enriched in the leaves of *Fagopyrum esculentum* (common buckwheat) (Li et al., 2019; Hou et al., 2021). Flavonoid components are identified in *Citrus* fruits, naringin in *Citrus maxima*, eriocitrin in *Citrus limon* and hesperidin in *Citrus sinensis* (Wang S. et al., 2017). 13 flavonoid components are found in different tissues of *Citrus reticulata*, including poncirin, didymin, neohesperdin, hesperidin, naringin, narirutin, 5-demethylnobiletin, tangeretin, nobiletin, sinensetin, isosinensetin, eriocitrin and neoeriocitrin (Zhao et al., 2021). The abundant flavonoid components, including isocarthamidin-7-O-glucuronide, apigenin-7-O-glucuronide, chrysin-7-O-glucuronide, chrysin, apigenin, scutellarin, baicalin and baicalein are contained in the shoots of *Scutellaria baicalensis* (Song et al., 2020). In addition, 19 flavonoid substances also are identified in the flower of *S. baicalensis* (Song et al., 2020). Flavonoid components are ubiquitous in *A. membranaceus* tissues, including roots, stems, leaves, fruits, seeds and flowers. At present, 52 flavonoid components, including baicalin, isoquercitrin, 3-*O*-*β*-*D*-glc-isorhamnetin, 7-*O*-*β*-*D*-glc-pratensein and 7-*O*-methylisomucronulatol, are identified in *A. membranaceus* (Dungérdorzh et al., 1974; Subarnas et al., 1991; Shirataki et al., 1997; Lin et al., 2000; Ju et al., 2008; Pei et al., 2008; Zheng et al., 2011; Wang et al., 2014; Xi et al., 2015).

Flavonoids have a variety of chemical structures, but they share the same basic skeleton in the early stages of formation (Singla et al., 2019; Shen et al., 2022). The basic skeleton mainly contains three rings (C6-C3-C6) (Singla et al., 2019; Shen et al., 2022). In general, flavonoids are mainly divided into seven classification based on chemical structure differences, including flavones, isoflavones, flavonols, flavanols, flavanones, chalcones and anthocyanidins (Shen et al., 2022). The flavonoid components are derived from the acetate pathway and shikimate pathway and then are modified to form different derivatives (Koirala et al., 2016; Tohge et al., 2017). Phenylalanine as an initial product is catalysed by phenylalanine ammonialyase catalysis to cinnamic acid in the shikimate pathway (Shen et al., 2022). Subsequently, the C4 position of cinnamic acid is hydroxylated by cinnamic acid hydroxylase to produce p-coumaric acid (Im et al., 2021). Coumaric acid is catalyzed by coumarin CoA ligase to produce synthesised p-coumaroyl CoA (Santín and Moncalián, 2018). Meanwhile, malonyl CoA plays a crucial role in the extension of long-chain fatty acids and the production of flavonoids (Santín and Moncalián, 2018). Naringenin chalcone is produced by chalcone synthase with p-coumaroyl CoA and malonyl CoA as substrate (Santín and Moncalián, 2018). Naringenin chalcone undergoes catalyzed by chalcone isomerase to produce flavanones (Tohge et al., 2017). In the flavonoid biosynthesis pathway, naringenin is used as the common precursor of end products and most intermediate metabolites. Naringenin generates flavones through flavone synthase I or flavone synthase II, and isoflavones through isoflavone synthase (Lukačin et al., 2001; Martens et al., 2003). Naringenin can react with flavanone-3-hydroxylase to catalyze the formation of dihydrokaempferol (Charrier et al., 1995; Pelletier and Shirley, 1996a). Meanwhile, naringenin is the substrate catalyzed by flavonol 3′5′-hydroxylase and flavonol 3′-hydroxylase to dihydromyricetin and dihydroquercetin, respectively (Vetten et al., 1999). Dihydroflavonol can be converted into flavonols under the action of the influence of flavonol synthase (Lukacin et al., 2003; Turnbull et al., 2004). The flavonoid biosynthesis pathway is diverse and complicated, and a series of enzymes play an important role in the metabolic pathway.

At present, the strategy of detecting substance content and gene expression during plant growth and development has been widely applied to study the molecular mechanism of flavonoid synthesis. In *C. reticulata*, flavonoid content and RNA-seq are measured at eight stages of fruit development, illustrating transcriptional regulation of flavanones and flavones. In addition, the *CitCHIL1* gene enhances the accumulation of flavanones and flavones, and is regulated by CitERF32 and CitERF33 transcription factors (Zhao et al., 2021). With the publication of the genome of *Lonicera japonica*, dynamic gene expression changes provide new insights into the evolution and dynamic fluctuation of flower colour in plants. This study reveals the associated expression of biosynthetic genes associated with the carotenoid accumulation and demonstrates that carotenoid degradation plays an important role in the dynamic flower colouration of *L. japonica* (Pu et al., 2020). The key genes of flavonoid biosynthetic (*PAL*, *4 C L*, *CHS*, *GT*, and *RT*) are identified during *Anoectochilus roxburghii* development (Guo, 2020). This strategy also has applications in fruit development, pigment accumulation, and abiotic stress.

The major components of *A. membranaceus* include flavonoids, saponins and polysaccharides, which are present in all tissues simultaneously (Auyeung et al., 2016; Liang et al., 2020). In *A. membranaceus* roots, flavonoid content and RNA-seq are measured at five different developmental stages, including vegetative, florescence, fruiting, fruit ripening and defoliating stages. A total of 86 genes related to flavonoid, isoflavonoid and phenylpropanoid synthesis are identified in transcriptome sequencing (Liang et al., 2020). The flavonoid content is the lowest at the fruiting stage and continuously increases from fruiting to defoliating in the roots of *A.* membranaceus (Liang et al., 2020). In this study, the flavonoid content continuously decreased from DAF5 to DAF30 stages in the fruits of *A. membranaceus*. The regular change of flavonoid content is more helpful to explain the transcriptional regulation of flavonoid biosynthesis in *A. membranaceus*. This study improves understanding of transcriptional regulation of flavonoid biosynthesis and the key genes of flavonoid biosynthetic are identified in *A. membranaceus*. Our results not only deepen the understanding of the flavonoid synthesis mechanism but also provided useful resources for future studies on the high flavonoid molecular breeding of *A. membranaceus*.

## Materials and methods

### Plant materials

*A. membranaceus* grew in a natural environment with vigorous growth and a normal fruit setting. The samples of the *A. membranaceus* were collected at six fruit different developmental stages, DAF5 (5 days after the flower), DAF10 (10 days after the flower), DAF15 (15 days after the flower), DAF20 (20 days after the flower), DAF25 (25 days after the flower) and DAF30 (30 days after the flower). All of the samples were preserved in liquid nitrogen. Three biological replicates were performed in the fruit tissue at six developmental stages. As described previously, a portion of the samples was used for the determination of flavonoids (Liang et al., 2020), and the other portion of the sample was extracted with total RNA for transcriptome sequencing (Wang et al., 2009; Jiang et al., 2011).

### RNA-seq analysis

For transcriptome profiling, the read count was the number of reads compared to the exon in high-throughput sequencing, and the count of all genes was calculated by HTseq-count software (Anders et al., 2015). The count was converted to Fragments Per Kilobase Million (FPKM) using the GenomicFeatures package included in R (https://www.r-project.org/). The gene expression levels in each sample were converted into a data matrix, and PCA was analyzed using the OmicShare tools, a free online platform for data analysis (https://www.omicshare.com/tools). A *p*-value of 0.05 and the absolute value of |log_2_ (Fold Change)| ≥ 1 were set as the threshold for significantly differential expression (Audic and Claverie, 1997). We pairwise compared six developmental stages in order to obtain differentially expressed genes (DEGs) based on the FPKM value. The shared DEGs were analyzed and extracted using UpSet included in the EVenn tool (Chen et al., 2021).

### Expression trend analysis

To investigate patterned differences in expression profiles during fruit developmental stages, we analyzed dynamic expression trends of genes using the OmicShare tools. Gene expression was normalized with log_2_(FC) and gene expression trends were set to 80 profiles using the Dynamic Trend Analysis tool with a *p*-value of significant expression trend < 0.05 and change multiple > 2. We extracted the expression of all genes in the significant profiles. Then, the average expression of genes in the profile was calculated at six fruit developmental stages. Based on the *p*-value and number of profiles, the significance profiles were combined using the cat command of the Linux system. The genes from the combined profiles were clustered according to gene expression using the pheatmap package included in R (https://www.r-project.org/). The cluster genes with modular genes were compared using the Interactive Venn diagram included in the EVenn tool (Chen et al., 2021).

### Enrichment analysis of genes within profiles

To understand gene function, we excavated function information based on the shared database. First, the cellular component, molecular function, and biological process of genes with the profile were annotated using Gene Ontology (GO) database (Ashburner et al., 2000). Then, pathways of the genes within the profile were enriched using the Kyoto Encyclopedia of Genes and Genomes (KEGG) database (Kanehisa and Goto, 2000). The terms/pathways of enrichment between profiles were compared, while the underlying function of genes within profiles was analyzed by the annotating information.

### Molecular mechanism of flavonoid-related genes analysis

To understand the molecular mechanism of *A. membranaceus* flavonoid-related genes, we first screened the annotating information of GO and KEGG. Flavonoid biosynthesis, isoflavonoid biosynthesis, and flavone and flavonol biosynthesis pathways were obtained by the KEGG database. Combined with the significantly enriched pathway, the flavonoid biosynthesis pathway in *A. membranaceus* was constructed based on flavonoid-related genes similar to flavonoid content during fruit developmental stages. The interaction network of flavonoid-related proteins was constructed according to the orthologs of *A. thaliana* using STRING (Pertea et al., 2015). We used Cytoscape software to visualize the network (Otasek et al., 2019). The UniProtKB tool was used for the symbol and description of flavonoid-related proteins (The UniProt, 2021). The expression patterns of flavonoid-related genes at six fruit developmental stages were demonstrated using the pheatmap package included in R (https://www.r-project.org/).

## Results

### Transcriptome divergence in fruit tissue at six developmental stages

**FIGURE 1:**
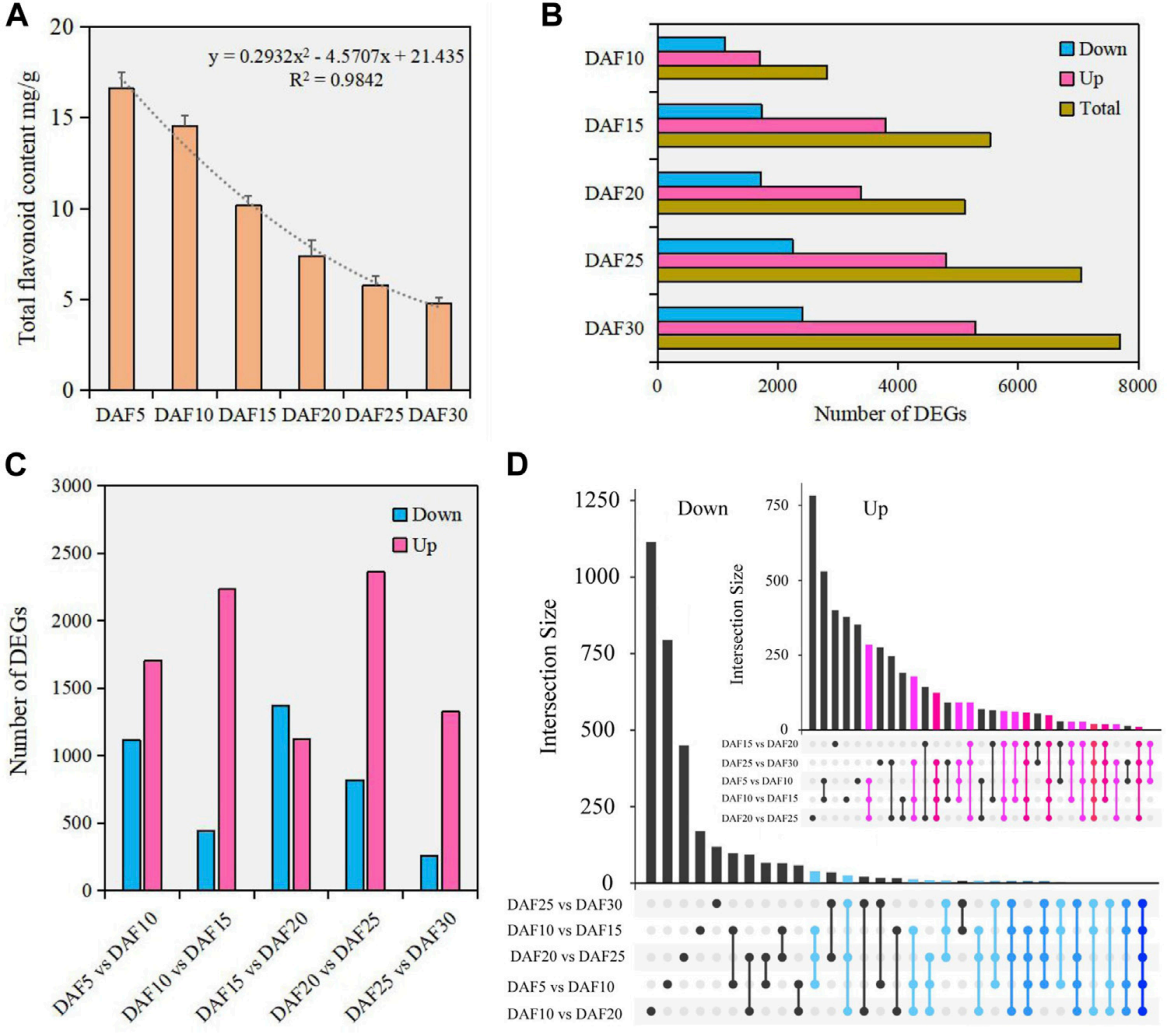
The analysis of flavonoids and DEGs in fruit tissue at six developmental stages. **(A)**. The content of flavonoids in fruit tissue at six developmental stages. **(B)**. The number of DEGs between DAF5 and other stages. **(C)**. The number of DEGs in adjacent developmental stages. **(D)**. The analysis of shared genes in adjacent developmental stages.

The accumulation and degradation of substances changed with the growth and development of plants. The content of total flavonoid was detected in fruit tissue of six developmental stages. Here, we found that the flavonoid content was the highest at the DAF5 stage and continuously decreased from DAF5 to DAF30 (Figure 1A). The flavonoid content in fruit tissue at the DAF5 stage was 3.5 times that in the DAF30 stage. The trend analysis showed that the flavonoid content decreased faster after the DAF15 stage. Therefore, the change rule of flavonoid content in *A. membranaceus* fruit development process was clearly visible.

To investigate the molecular mechanisms underlying the flavone metabolism of *A. membranaceus*, the RNA-seq of 18 samples were analyzed during fruit development. The PCA analysis data set was divided into 18 dimensions based on all gene expression levels in the samples. The variance contribution rate of the PC1 dimension was 41.5%, and that of the PC2 dimension was 22.6%. Pairwise comparison showed that the most DEGs were found between DAF5 and DAF30 stages, and the least DEGs were found between DAF25 and DAF30 stages. Meanwhile, in the comparison of DEGs between DAF5 and other stages, both the number of down-regulated and up-regulated DEGs showed an upward trend (Figure 1B). In adjacent developmental stages, the most DEGs were between DAF20 and DAF25 stages, including 2,362 up-regulated DEGs and 821 down-regulated DEGs (Figure 1C). The number of down-regulated DEGs was more than that of up-regulated DEGs only between DAF15 and DAF20 stages. Only 257 down-regulated DEGs were identified between DAF25 and DAF30 stages (Figure 1C). Twenty shared genes were identified in the up-regulated DEGs of five adjacent stages. Although the number of DEGs was large, the number of shared genes was relatively small in the down-regulated DEGs of five adjacent stages. (Figure 1D). The shared genes of up-regulated DEGs were clustered into five groups according to gene expression (Supplementary Figure S2). These shared genes were mainly highly expressed in DAF5 and DAF10 stages and hardly expressed in the DAF30 stage (Supplementary Figure S2). The molecular functions of shared genes were annotated as carbohydrate binding (GO: 0030246), protein kinase activity (GO: 0004672), ATP binding (GO: 0005524) and protein binding (GO: 0005515). These genes potentially play important regulatory roles in fruit development. The distribution of down-regulated DEGs indicated that most of these genes had the feature of time-specific expression and their molecular functions were performed at specific stages.

### Characterization of expression trend during fruit development

**FIGURE 2:**
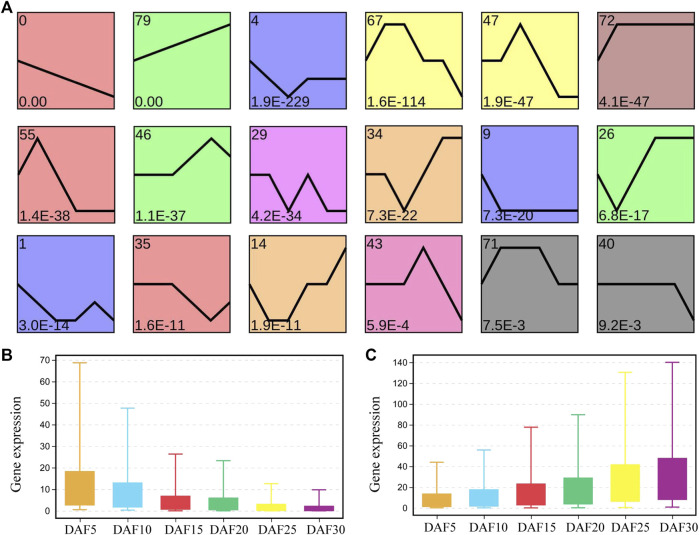
Analysis of gene expression trend in fruit tissue at six developmental stages. **(A)**. Profiles are ordered based on the *p*-value significance of gene assigned. **(B)**. The level of the gene expression at six developmental stages in profile 0. **(C)**. The level of gene expression at six developmental stages in profile 79.

To explore the possible expression pattern during six stages of fruit development in *A. membranaceus*, the expression trends of genes were performed to further divide the related genes into 80 profiles. Sixteen gene expression trends were significantly identified by screening with a minimum of 2-fold gene change (Figure 2A). We found that the profiles with continuous upward and downward trends showed the most significance. A total of 8,218 genes were present in these sixteen profiles, 2,974 in profile 0, 1,396 in profile 79 and 448 in profile 4 (Supplementary Figure S3). There were 2.1 times as many genes from profile 0 as from profile 79, and 33 times as from profile 43. Profile 0 and profile 79 account for 53.2% of the total genes in significant profiles. It could be seen that the genes with the rule of expression change were mainly concentrated in profile 0 and profile 79. The average expression level of genes from profile 0 and profile 79 was calculated at six developmental stages (Figures 2B and 2C). In profile 0, the average gene expression of DAF5 was 1.4 times and 7.6 times higher than that of DAF10 and DAF30, respectively. In adjacent developmental stages, the change multiple of average gene expression was the largest between DAF10 and DAF15. In profile 79, the average gene expression of DAF30 was 1.2 times and 6.6 times higher than that of DAF25 and DAF5, respectively. The change multiple of the average gene expression was 1.5 in the adjacent group, and the largest change multiple was 1.7 between DAF25 and DAF30. The average gene expression at all stages was more than twice as high in profile 79 as in profile 0.

**FIGURE 3:**
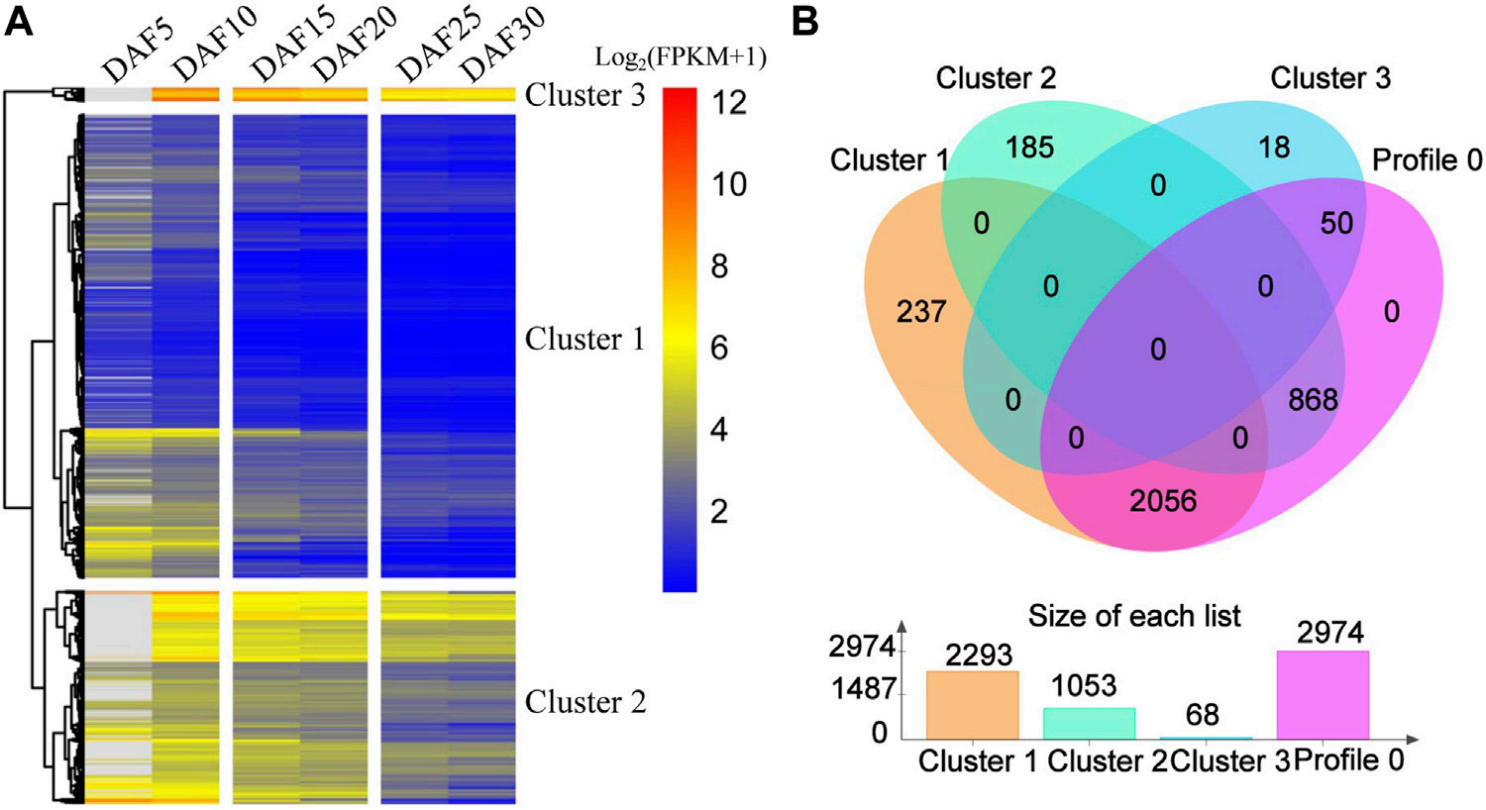
Comparative analysis of genes from profile 0 and cluster genes. **(A)**. Cluster analysis of expression trend genes from profile 0, profile 55 and profile 35. **(B)**. The Venn diagram represents the shared and unique genes among the profiles; The histogram represents the number of genes in profile 0, cluster 1, cluster 2 and cluster 3.

On the whole, profile 0, profile 55 and profile 35 showed a similar downward trends, and profile 79, profile 46 and profile 26 showed a similar upward trends (Figure 2A, Supplementary Figure S3). The expression of 259 genes showed an upward trend from DAF5 to DAF10 stage and a stable trend from DAF25 to DAF30 stage in profile 55. The expression of 181 genes showed a stable trend from DAF5 to DAF10 stage and an upward trend from DAF25 to DAF30 stage in profile 35. In the upward trend profiles, the gene expression showed a downward trend from DAF25 to DAF30 stage in profile 46 and from DAF5 to DAF10 stage in profile 26, respectively. Here, 3,414 genes from profile 0, profile 55 and profile 35 were clustered based on gene expression, and divided into three clusters (Figure 3A). Cluster 3, cluster 2 and cluster 1 showed high, medium and low expression, respectively. 2,293, 1,053 and 68 genes were divided into cluster 1, cluster 2 and cluster 3, respectively (Figure 3B). 2,056 shared genes were found between profile 0 and cluster 1. Meanwhile, shared genes were identified between profile 0 and others clusters. These results suggested that gene expression trends were not related to gene expression levels. Genes with different levels of expression played a role in fruit development.

### Functional enrichment of the profile genes

**FIGURE 4:**
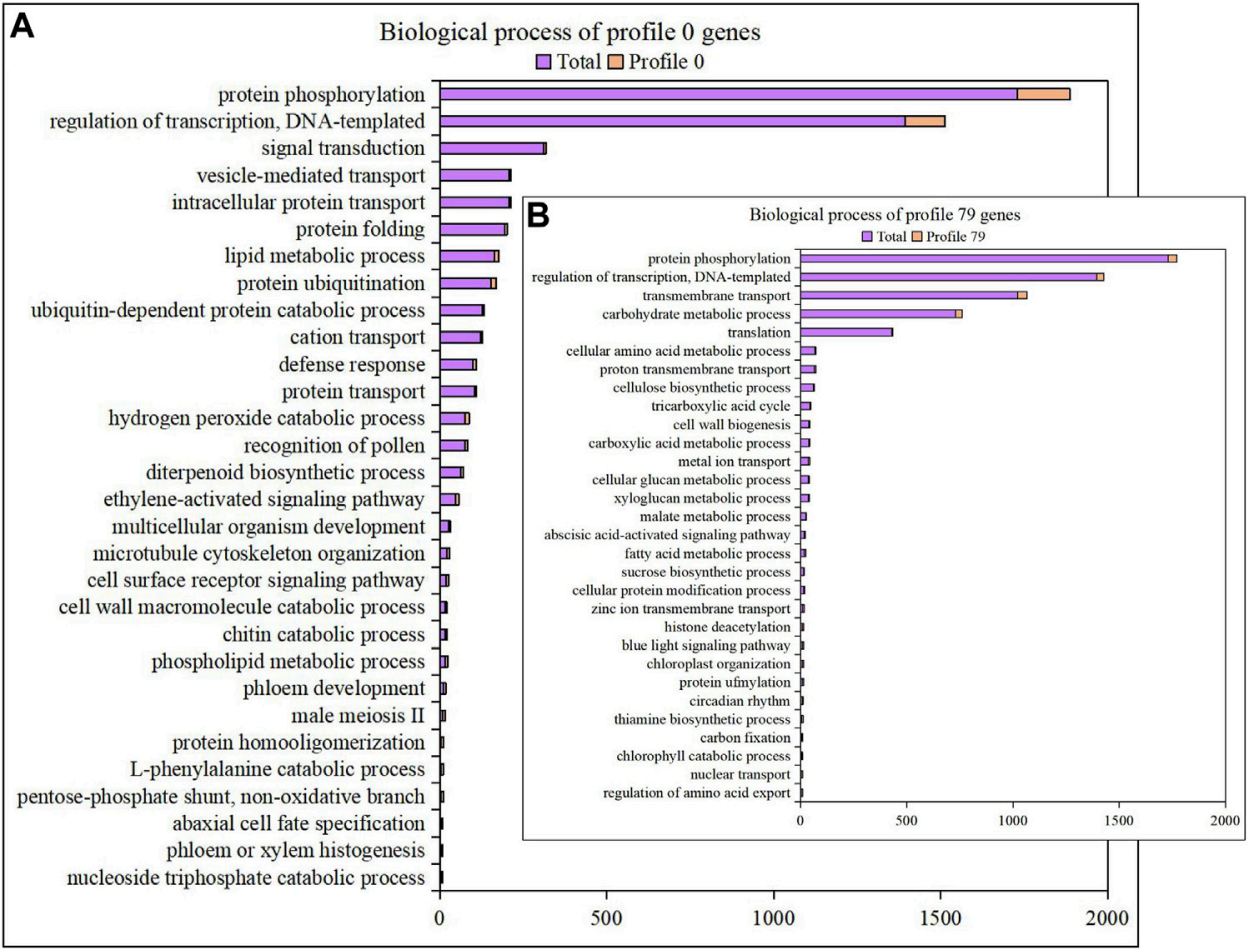
Functional enrichment of the profile genes. **(A)**. Gene enrichment with biological process of GO in profile 0. **(B)**. Gene enrichment with biological process of GO in profile 79.

We focused on profile 0 and profile 79 which were significantly associated with downward and upward trend for functional enrichment analysis. In profile 0, 271, 1,328 and 831 genes were annotated in GO of cellular component, molecular function and biological process terms, respectively (Supplementary Table S1). 45, 8 and 80 genes were annotated to the nucleus, extracellular region and membrane, respectively. Molecular functions involved nucleic acid binding (40 genes), protein kinase activity (157 genes), hydrolase activity, acting on ester bonds (28 genes), DNA-binding transcription factor activity (81 genes), enzyme inhibitor activity (18 genes) and endopeptidase inhibitor activity (8 genes). 25 biological processes were enriched in profile 0, including protein phosphorylation, regulation of transcription, DNA-templated and signal transduction (Figure 4A and Supplementary Table S1). In profile 79, 157, 718 and 446 genes were annotated in GO of cellular component, molecular function and biological process terms, respectively (Supplementary Table S2). GO of cellular component mainly included SAGA complex (4 genes), membrane (52 genes), nucleus (9 genes), cytoplasm (13 genes) and integral component of membrane (38 genes). Molecular function mainly included nucleic acid binding (21 genes), UDP-glycosyltransferase activity (14 genes), catalytic activity (51 genes), pyridoxal phosphate binding (14 genes) and ATP-dependent peptidase activity (9 genes). 29 biological processes were enriched in the profile 79, including protein phosphorylation, regulation of transcription, DNA-templated, transmembrane transport and carbohydrate metabolic process (Figure 4B and Supplementary Table S2). The shared GO terms included 8 terms belonging to the cellular component, 6 terms belonging to the molecular function and 2 terms belonging to the biological process in profile 0 and profile 79.

To understand the biological relevance of profile genes, biological pathways of gene involvement were enriched in profile 0 during six stages of fruit development. KEGG pathway enrichment analysis revealed numerous pathways related to genetic information processing, carbohydrate metabolism and biosynthesis of secondary metabolites (Supplementary Figure S4). Here, 4% of genes were involved in the biosynthesis of secondary metabolites, including flavonoid biosynthesis (map00941), isoflavonoid biosynthesis (map00943), flavone and flavonol biosynthesis (map00944) pathways (Supplementary Figure S5).

### Protein-protein interaction network of flavonoid-related proteins

**FIGURE 5:**
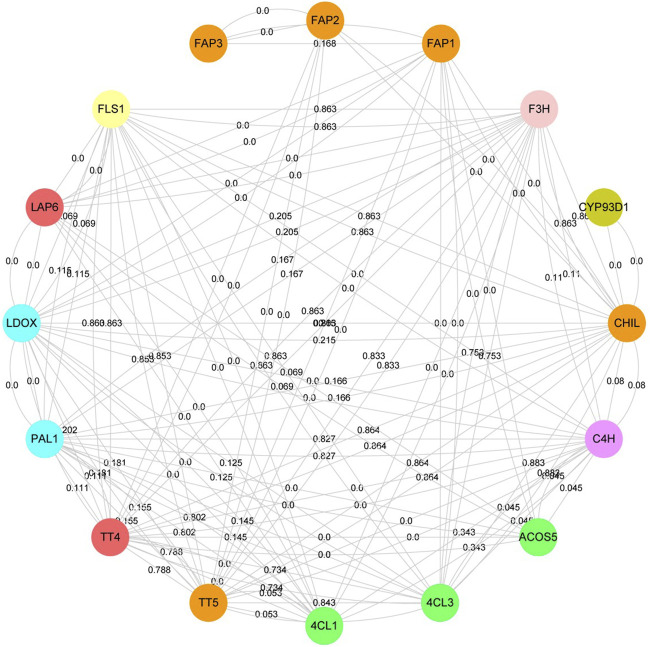
Protein-protein interaction network of flavonoid-related proteins. The same color represent that the proteins belong to the same protein family. The lines represent protein-protein interaction. The numbers on the lines represent gene co-expression.

To understand the molecular mechanism of flavonoid-related proteins, the interaction of flavonoid-related proteins was analyzed based on the STRING database (Figure 5, Supplementary Table S3). Flavonoid-related proteins were identified according to *A. thaliana* paralogous proteins, including phenylalanine ammonia-lyase (PAL), cinnamic acid 4-hydroxylase (C4H), 4-coumarate--CoA ligase (4CL), chalcone synthase (CHS), chalcone--flavanone isomerase (CHI), flavonol synthase/flavanone 3-hydroxylase (FLC), naringenin, 2-oxoglutarate 3-dioxygenase (F3H), flavone synthase (FNS) and 2-hydroxyisoflavanone synthase (IFS). 126 interacting proteins were identified among 16 flavonoid-associated proteins, of which 78 pairs were high confidence interacting proteins (Minimum required interaction score >0.700). Here, CYP93D1, belonging to the cytochrome P450 family interacted only with CHIL. TT4, TT5, FLS1, CHIL, and LDOX interacted with eleven proteins in this network, respectively. TT5, CHIL and FAPs belonged to the CHI family protein. However, we found that FAP3 interacted only with FAP1 and FAP2, and FAP1 and FAP2 interacted with seven and two proteins, respectively. These results indicated that CHI family members played different roles in the flavonoid biosynthesis pathway. The genes of 66.7% interacting protein belonged to co-expressive genes. Overall, the higher the confidence of protein-protein interaction, the higher the chance of gene co-expression.

### The molecular mechanism of flavonoid during fruit development in A. *membranaceus*

**FIGURE 6:**
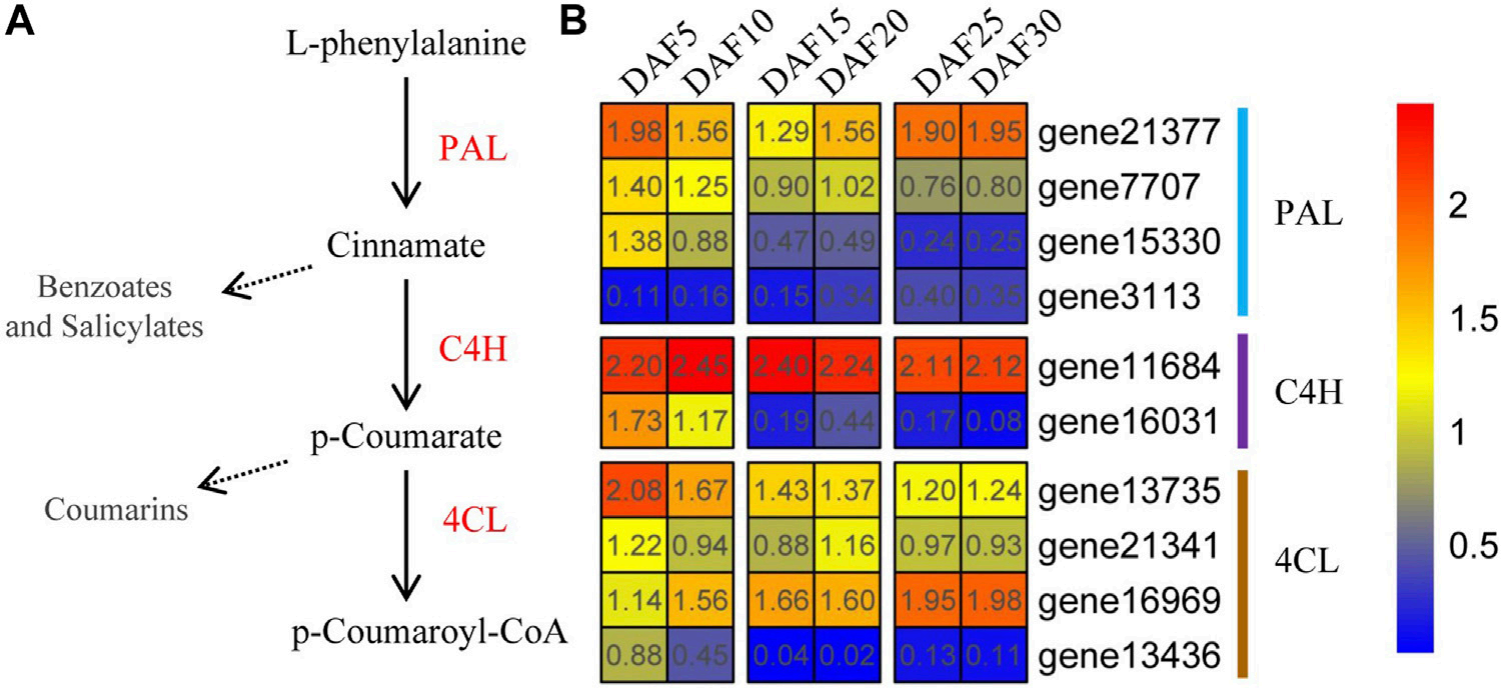
The formation of p-Coumaroyl-CoA pathway in the general phenylpropane pathway and expression of core genes. **(A)**. The formation of p-Coumaroyl-CoA pathway in the general phenylpropane pathway. **(B)**. Expression of core formation of p-Coumaroyl-CoA pathway genes at six fruit developmental stages. The quantitative values are processed with log_2_ (FPKM+1).

Previous studies have shown that the general phenylpropane pathway has essentially been completely elucidated and is a key step in the production of p-Coumaroyl-CoA (Figure 6A and Supplementary Table S4). PAL, a key enzyme in plant metabolism, catalyzed L-phenylalanine in a wide variety of natural products. The first reaction involving the PAL enzyme could catalyze L-phenylalanine into cinnamate based on the phenylpropane skeleton. Coumarate undergoes transformations catalyzed by C4H hydroxylase to produce p-Coumarate. p-Coumarate is converted to form phytoene by 4CL ligase, which is involved in the last step of the general phenylpropanoid pathway. Here, Four *AmPAL*, two *AmC4H* and four *Am4CL* genes were found in *A. membranaceus*, respectively. We found that four genes (*AmPAL2*, *AmPAL3*, *AmC4H2*, and *Am4CL1*) belonged to profile 0. The expression of *AmPAL* genes showed three expression trends during fruit development (Figure 6B). The expressions of *AmPAL2* and *AmPAL3* genes in the DAF5 stage were the highest, which were 4.6 and 30 times that in the DAF30 stage, respectively. The expression of the *AmC4H2* gene showed a downward trend but increased slightly in the DAF20 stage. The expression of the *AmC4H2* gene in the DAF5 stage was 3.8 times and 279 times higher than that in the DAF10 stage and DAF30 stage, respectively. The expression trend of the *Am4CL3* gene was contrary to that of the *Am4CL1* and *Am4CL4* genes, showing an upward expression trend. *AmPAL2*, *AmPAL3*, *AmC4H2*, *Am4CL1* and *Am4CL4* mRNA levels were reduced during DAF5 to DAF30 stages of *A. membranaceus* fruit development. Combined with the characteristics of flavonoid content changes, it suggested that these genes played an important role in the formation of p-Coumaroyl-CoA in the general phenylpropane pathway.

**FIGURE 7:**
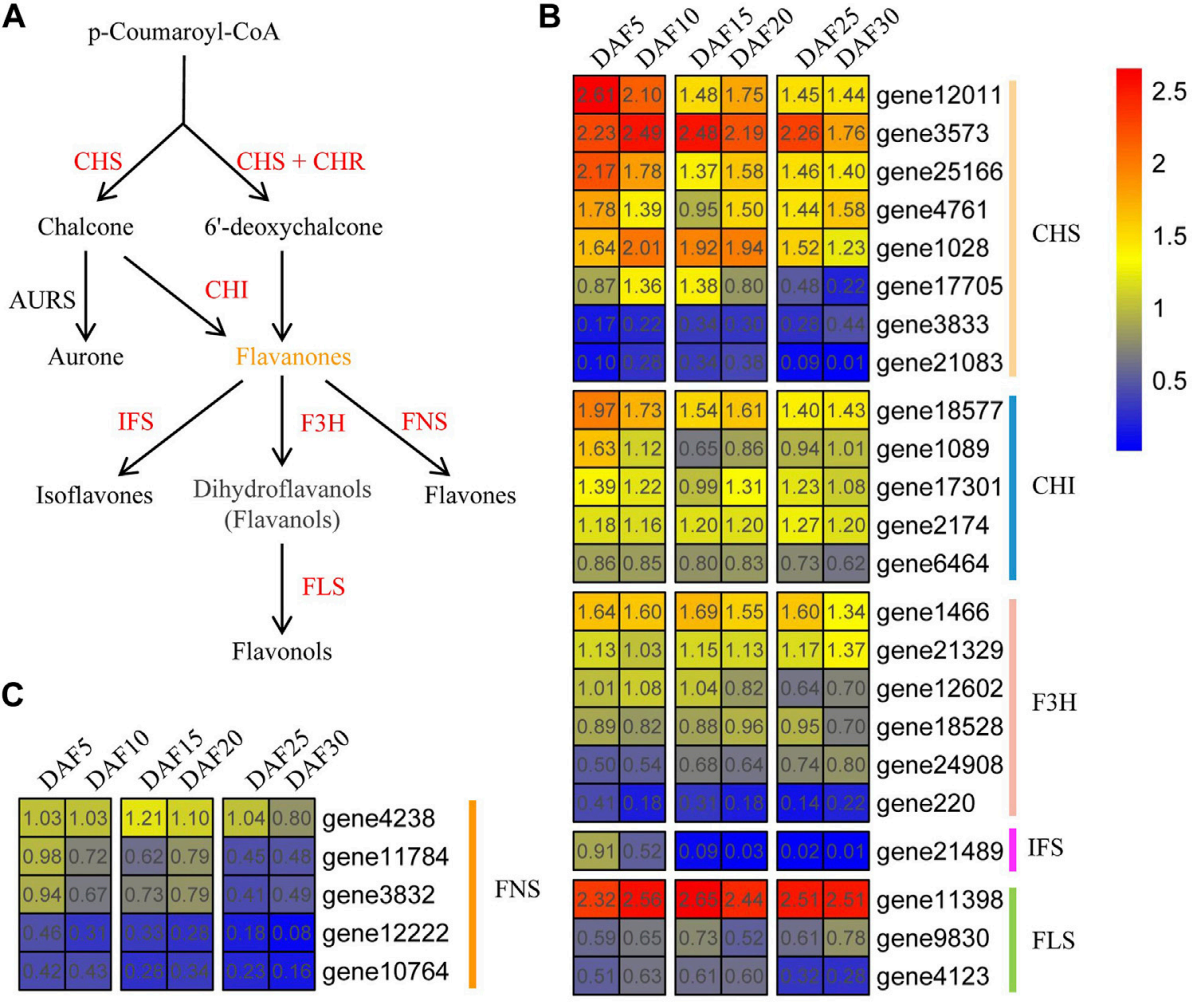
Flavonoid biosynthesis pathway and expression of core genes. **(A)**. Part of flavonoid biosynthesis pathway. **(B,C)**. Expression of core flavonoid biosynthesis pathway genes at six fruit developmental stages. The quantitative values are processed with log_2_ (FPKM+1).

As a link, p-Coumaroyl-CoA plays a crucial role in flavonoid biosynthesis. First, p-Coumaroyl-CoA is catalyzed to form chalcone and 6′-deoxychalcone by CHS synthase, respectively. Chalcone and 6′-deoxychalcone then are catalyzed by CHI isomerase to produce flavonoids (Figure 7A and Supplementary Table S4). Here, Eight *AmCHS*, five *AmCHI*, six *AmF3H*, one *AmIFS*, three *AmFLS* and four *AmFNS* genes were found in *A. membranaceus*, respectively (Figures 7B and 7C). Five genes (*AmCHS1*, *AmCHI1*, *AmFNS4*, *AmFNS5*, and *AmIFS*) belonged to profile 0, and one gene (*AmF3H3*) belonged to profile 35. The expression trend of the *AmCHS* gene did not show a continuous upward or downward trend. Such as, the gene expression of *AmCHS1*, *AmCHS3* and *AmCHS4* genes was up-regulated from DAF15 to DAF20 stage. The gene expression of *AmCHS2* and *AmCHS6* genes were down-regulated from DAF15 to DAF20 stage. Overall, *AmCHS* genes were low expressed in the DAF30 stage. The expression trends of *AmCHS* and *AmF3H* genes were similar to that of *AmCHS* genes. The expressions of *AmCHI1*, *AmCHI2* and *AmCHI3* genes in the DAF5 stage were the highest, which were 3.6, 4.6 and 2.1 times that in the DAF30 stage, respectively. Compared with the DAF5 stage, *AmF3H1*, *AmF3H3*, *AmF3H4* and *AmF3H5* genes were down-regulated, and *AmF3H2* and *AmF3H6* genes were up-regulated in the DAF30 stage. The expression of the *AmIFS* gene showed a downward trend and almost no expression after the DAF15 stage. The *AmFLS1* gene had high expression levels at all stages, whereas the *AmFLS2* and *AmFLS3* genes were just the opposite. The expression of the *AmFLS3* gene was up-regulated from DAF5 to DAF10 and down-regulated from DAF10 to DAF30. Globally, the expression of *AmFNS* genes showed a downward trend during fruit developmental stages. Combined with the characteristics of flavonoid content changes, it suggested that these down-regulated genes played an important role in the flavonoid biosynthesis pathway.

## Discussion

Flavonoids are widely distributed in plants as secondary metabolites. Flavonoids are identified in a large number of plants, such as *Oryza sativa* and *Glycine max* in crops (Jayachandran and Xu, 2019; Sen et al., 2020), *Lycopersicon esculentum* and *Brassica rapa* in vegetables (Koo et al., 2001; Da Silva Souza et al., 2020), *Malus domestica* and *Vitis vinifera* in fruits (Espley et al., 2019; Torres et al., 2020), and *Salvia miltiorrhiza* and *Rhodiola rosea* in medicinal plants (Jiang et al., 2020; Döring et al., 2022). The flavonoid content and component were significantly different among different plant species. The results of flavonoid determination of 62 tropical plants show the highest total flavonoids content is 2.72 mg/g in *Allium cepa*, followed by *Azadirachta Indica* (2.04 mg/g), *Capsicum frutescens* (1.66 mg/g), *Camellia sinensis* (1.49 mg/kg) (Koo et al., 2001). *O. sativa*, as the staple food and model organism, contains flavonoids such as flavone O-glycosides, acylated flavonoid O-glycosides, tricin-O-glycosides and flavonol-O-glycosides (Yan et al., 2019). The major flavonoid in extracts of *Zea mays* silk is luteolin, followed by apigenin and formononetin (Hu et al., 2020). In this study, the flavonoid content was the highest at the DAF5 stage (16.6 mg/g) during fruit developmental stages and continuously decreased from DAF5 to DAF30. In *C. reticulata* and *Ziziphus jujuba*, flavonoid content also continuously decreased during fruit development (Xue et al., 2021; Zhao et al., 2021). The flavonoid content is the lowest at the fruiting stage and continuously increases from fruiting to defoliating in the roots of *A.* membranaceus (Liang et al., 2020). The variation of flavonoid content does not show regular changes, which may be attributed to the difference between the growth rule of the aboveground part and the underground part of the plant. Our study showed that flavonoid content continuously decreased during fruit development, and this regular change of flavonoid content is more helpful to explain the transcriptional regulation of flavonoid biosynthesis in *A. membranaceus*.

The mechanism of flavonoid synthesis and identification of regulatory genes has been an important and hot issue in medicinal plants. With the rapid development of the next generation sequencing, RNA sequencing (RNA-seq) is widely used in the analysis of transcriptomes of various organisms. The key genes of stilbene, flavonoid and anthraquinone biosynthesis are identified by tissue-specific transcriptome in the medicinal plant *Polygonum cuspidatum* (Wang et al., 2021). The mechanism of anthocyanin biosynthesis is analyzed using metabolomics and transcriptomics in *S. miltiorrhiza* (Liang et al., 2020). In our study, we used a combination of substance content and transcriptome to elucidate the molecular mechanism of flavonoids during fruit development. Meanwhile, many plants, such as *L. japonica*, *Lycium barbarum* and *M. domestica*, have studied the corresponding molecular mechanism through changes in substance content and gene expression during development (Wang C. et al., 2020; Pu et al., 2020). These studies not only greatly deepened understanding the mechanism of substance synthesis and degradation, in the same time, it laid an important foundation to molecular regulation of fruit development improvement. The strategy of gene expression trend is an effective means to explain the scientific problems of the plant development process and continuously changing phenotype. *L. japonica* has the characteristic of dynamic flower colouration, which changes from white to gold during flower development. The molecular mechanism of dynamic flower colouration is revealed based on the carotenoid content and gene expression trend during six different flower developmental stages (Pu et al., 2020). In our study, a total of sixteen significant profiles were identified during six different fruit developmental stages. The gene expression trend in profile 0 was positively correlated with flavonoid content, while the gene expression trend in profile 79 was negatively correlated with flavonoid content at six developmental stages. We hypothesized that the genes in profile 0 were positively regulating the synthesis of flavonoids, while the genes in profile 79 were negatively regulating the synthesis of flavonoids.

The gene expression characteristic of space and time at the transcriptional level regulates plant phenotype, substance content and stress response. Twenty-seven flavonoid-related genes are screened in the fruit, root, and leaf tissues of *Alpinia oxyphylla*, of which seven genes are highly expressed in fruits and six genes are highly expressed in roots (Yuan et al., 2021). The five *CHS* genes are identified in three different tissues of *Dendrobium officinale* based on the gene expression and flavonoid content (Yuan et al., 2020). In *A. membranaceus*, we found that ten genes were involved in the general phenylpropane pathway, and twenty-seven genes were involved flavonoid biosynthesis pathway. On the whole, flavonoid-related genes showed the highest expression level at the early stage of fruit development. The expression level of the *CsCHI* genes was highly expressed after the second physiological fruit-falling period in *Citrus sinensis* (Wan et al., 2022)*.* The expression level of the *CitFAP2* gene is the highest at 120 days after flowering, while the expression of *CitCHI1*, *CitCHIL1/2* and *CitFAP1/3* genes reach the peak at 30 days after flowering (Zhao et al., 2021). The expression patterns of flavonoid-related genes are gene expression characteristic of space and time at the transcriptional level.

The flavonoid biosynthesis pathway is diverse and complicated, which requires the phenylpropanoid biosynthesis pathway to provide a substrate (Shen et al., 2022). The flavonoid biosynthesis, phenylpropanoid biosynthesis and anthocyanin biosynthesis pathways are enriched during three different developmental stages in *Lycium barbarum* and *Lycium ruthenicum* (Wang C. et al., 2020). In our study, the expression level of genes involved in the general phenylpropane pathway was higher than that of genes involved in the flavonoid biosynthesis pathway. In contrast, *AmFNS* genes, which were the downstream genes of the flavonoid pathway, had a lower gene expression. We speculated that this result might be directly related to the low content of isoflavones in *A. membranaceus*. A series of enzymes play an important role in the flavonoid biosynthesis pathway. Studies prove that several key enzyme genes, including *CHS*, *CHI*, *F3′H*, and *DFR*, mediate flavonoid compound synthesis by *A. thaliana* mutants (Jackson et al., 1995; Pelletier and Shirley, 1996b; Yuan et al., 2007; Jiang et al., 2015). The expression of the *CitCHIL1* gene is positively correlated with flavonoid content and enhances the accumulation of flavanones and flavones in *C. reticulata* (Zhao et al., 2021). The key genes involved in the flavonoid biosynthesis pathway have been identified in *Malus sieversii*, *Artemisia annua*, *G. biloba* and other plants (Wang N. et al., 2017; Liu et al., 2020; Qin et al., 2021). Taken together, *PAL*, *C4H*, *4CL*, *CHS*, *CHI*, *F3H*, *IFS*, *FNS* and *FLS* genes play a crucial role in the flavonoid biosynthesis pathway.

## Conclusion

In the present study, we analyzed the association between flavonoid content and gene expression pattern during six different fruit developmental stages. A total of 37 genes involved in flavonoid synthesis were identified in *A. membranaceus*. The expression pattern of flavonoid-related genes was highly correlated with flavonoid content. These genes might potentially be involved in flavonoid biosynthesis in fruit of *A. membranaceus*. Our results not only provided useful resources for future studies on the high flavonoid molecular breeding of *A. membranaceus*, but also laid a foundation for the production of flavonoids for the treatment of cardiovascular diseases, viral myocarditis and other diseases in the future.

## Data Availability

The datasets presented in this study can be found in online repositories. The names of the repository and link to the data can be found below: Figshare; https://figshare.com/articles/dataset/_/20098925.

## References

[B1] Anders S. Pyl P. T. Huber W. (2015). HTSeq--a Python framework to work with high-throughput sequencing data. Bioinformatics 31 (2), 166–169. 10.1093/bioinformatics/btu638 25260700 PMC4287950

[B2] Ashburner M. Ball C. A. Blake J. A. Botstein D. Butler H. Cherry J. M.fnm (2000). Gene ontology: Tool for the unification of biology. The gene Ontology consortium. Nat. Genet. 25(1), 25–29. 10.1038/75556 10802651 PMC3037419

[B3] Audic S. Claverie J.-M. (1997). The significance of digital gene expression profiles. Genome Res. 7 (10), 986–995. 10.1101/gr.7.10.986 9331369

[B4] Auyeung K. K. Han Q.-B. Ko J. K. (2016). Astragalus membranaceus: A review of its protection against inflammation and gastrointestinal cancers. Am. J. Chin. Med. 44 (01), 1–22. 10.1142/S0192415X16500014 26916911

[B5] Bratkov V. M. Shkondrov A. M. Zdraveva P. K. Krasteva I. N. (2016). Flavonoids from the genus Astragalus: Phytochemistry and biological activity. Pharmacogn. Rev. 10 (19), 11–32. 10.4103/0973-7847.176550 27041870 PMC4791984

[B6] Charrier B. Coronado C. Kondorosi A. Ratet P. (1995). Molecular characterization and expression of alfalfa ( *Medicago sativa* L.) flavanone-3-hydroxylase and dihydroflavonol-4-reductase encoding genes. Plant Mol. Biol. 29 (4), 773–786. 10.1007/bf00041167 8541503

[B7] Chen T. Zhang H. Liu Y. Liu Y.-X. Huang L. (2021). EVenn: Easy to create repeatable and editable Venn diagrams and Venn networks online. J. Genet. Genomics 48 (9), 863–866. 10.1016/j.jgg.2021.07.007 34452851

[B8] Chen Z. Liu L. Gao C. Chen W. Vong C. T. Yao P. (2020). Astragali Radix (huangqi): A promising edible immunomodulatory herbal medicine. J. Ethnopharmacol. 258, 112895. 10.1016/j.jep.2020.112895 32330511

[B9] Da Silva Souza M. A. Peres L. E. P. Freschi J. R. Purgatto E. Lajolo F. M. Hassimotto N. M. A. (2020). Changes in flavonoid and carotenoid profiles alter volatile organic compounds in purple and orange cherry tomatoes obtained by allele introgression. J. Sci. Food Agric. 100 (4), 1662–1670. 10.1002/jsfa.10180 31808163

[B10] Döring K. Langeder J. Duwe S. Tahir A. Grienke U. Rollinger J. M. (2022). Insights into the direct anti-influenza virus mode of action of *Rhodiola rosea* . Phytomedicine 96, 153895. 10.1016/j.phymed.2021.153895 35026524

[B11] Dungérdorzh D. Petrenko V. V. Deryugina L. I. (1974). Aglycone composition of the flavonoid glycosides of *Astragalus mongolicus* . Chem. Nat. Compd. 10 (2), 260. 10.1007/BF00563635

[B12] Espley R. V. Leif D. Plunkett B. McGhie T. Allan A. C. (2019). Red to Brown: An elevated anthocyanic response in apple drives ethylene to advance maturity and fruit flesh browning. Front. Plant Sci. 10, 1248. 10.3389/fpls.2019.01248 31649709 PMC6794385

[B13] Fu J. Wang Z. Huang L. Zheng S. Wang D. Chen S. (2014). Review of the botanical characteristics, phytochemistry, and pharmacology of *Astragalus membranaceus* (huangqi). Phytotherapy Res. 28 (9), 1275–1283. 10.1002/ptr.5188 25087616

[B14] Guo S. (2020). Combined metabolome and transcriptome analyses reveal the effects of mycorrhizal fungus ceratobasidium sp. AR2 on the flavonoid accumulation in *Anoectochilus roxburghii* during different growth stages. Int. J. Mol. Sci. 21, 564. 10.3390/ijms21020564 PMC7013922 31952330

[B15] Hou S. Du W. Hao Y. Han Y. Li H. Liu L. (2021). Elucidation of the regulatory network of flavonoid biosynthesis by profiling the metabolome and transcriptome in *tartary buckwheat* . J. Agric. Food Chem. 69 (25), 7218–7229. 10.1021/acs.jafc.1c00190 34151566

[B16] Hu X. Liu J. Li W. Wen T. Li T. Guo X.-B. (2020). Anthocyanin accumulation, biosynthesis and antioxidant capacity of black sweet corn (*Zea mays* L.) during kernel development over two growing seasons. J. Cereal Sci. 95, 103065. 10.1016/j.jcs.2020.103065

[B17] Im K. J. Christopher H. S. Bonawitz N. D. Franke R. B. Clint C. (2021). Spatio-temporal control of phenylpropanoid biosynthesis by inducible complementation of a cinnamate 4-hydroxylase mutant. J. Exp. Bot. 72 (8), 8. 10.1093/jxb/erab055 33585900

[B18] Jackson J. A. Fuglevand G. Brown B. A. Shaw M. J. Jenkins G. I. (1995). Isolation of *Arabidopsis* mutants altered in the light-regulation of chalcone synthase gene expression using a transgenic screening approach. Plant J. 8 (3), 369–380. 10.1046/j.1365-313X.1995.08030369.x 7550375

[B19] Jayachandran M. Xu B. (2019). An insight into the health benefits of fermented soy products. Food Chem. 271 (15), 362–371. 10.1016/j.foodchem.2018.07.158 30236688

[B20] Jiang L. Schlesinger F. Davis C. A. Zhang Y. Li R. Salit M. (2011). Synthetic spike-in standards for RNA-seq experiments. Genome Res. 21 (9), 1543–1551. 10.1101/gr.121095.111 21816910 PMC3166838

[B21] Jiang T. Zhang M. Wen C. Xie X. Liu L. (2020). Integrated metabolomic and transcriptomic analysis of the anthocyanin regulatory networks in *Salvia miltiorrhiza* Bge. flowers. BMC Plant Biol. 20 (1), 349. 10.1186/s12870-020-02553-7 32703155 PMC7379815

[B22] Jiang W. Yin Q. Wu R. Zheng G. Liu J. Dixon R. A. (2015). Role of a chalcone isomerase-like protein in flavonoid biosynthesis in *Arabidopsis thaliana* . J. Exp. Bot. 66 (22), 7165–7179. 10.1093/jxb/erv413 26347569 PMC4765788

[B23] Ju S. K. Min H. Y. Lee E. J. Sam S. K. (2008). Phytochemical studies on *Astragalus* root(1) - saponins. Nat. Product. Sci. 14 (1), 37–46.

[B24] Kanehisa M. Goto S. (2000). Kegg: Kyoto encyclopedia of genes and genomes. Nucleic acids Res. 28 (1), 27–30. 10.1093/nar/28.1.27 10592173 PMC102409

[B25] Koirala N. Thuan N. H. Ghimire G. P. Thang D. V. Sohng J. K. (2016). Methylation of flavonoids: Chemical structures, bioactivities, progress and perspectives for biotechnological production. Enzyme Microb. Technol. 86, 103–116. 10.1016/j.enzmictec.2016.02.003 26992799

[B26] Li J. Yang P. Yang Q. Gong X. Ma H. Dang K. (2019). Analysis of flavonoid metabolites in buckwheat leaves using UPLC-ESI-MS/MS. Mol. (Basel, Switz. 24 (7), 1310. 10.3390/molecules24071310 PMC6479795 30987158

[B27] Li X. Qu L. Dong Y. Han L. Liu E. Fang S. (2014). A review of recent research progress on the astragalus genus. Mol. (Basel, Switz. 19 (11), 18850–18880. 10.3390/molecules191118850 PMC6270929 25407722

[B28] Liang J. Li W. Jia X. Zhang Y. Zhao J. (2020). Transcriptome sequencing and characterization of *Astragalus membranaceus* var. mongholicus root reveals key genes involved in flavonoids biosynthesis. Genes & Genomics 42 (8), 901–914. 10.1007/s13258-020-00953-5 32519170

[B29] Lin L. Z. He X. G. Lindenmaier M. Nolan G. Cordell G. A. (2000). Liquid chromatography-electrospray ionization mass spectrometry study of the flavonoids of the roots of *Astragalus mongholicus* and *A. membranaceus* . J. Chromatogr. A 876 (1-2), 87–95. 10.1016/s0021-9673(00)00149-7 10823504

[B30] Liu P. Zhao H. Luo Y. (2017). Anti-Aging implications of *Astragalus membranaceus* (huangqi): A well-known Chinese tonic. Aging Dis. 8 (6), 868–886. 10.14336/AD.2017.0816 29344421 PMC5758356

[B31] Liu S. Wang L. Cao M. Pang S. Li W. Kato-Noguchi H. (2020). Identification and characterization of long non-coding RNAs regulating flavonoid biosynthesis in *Ginkgo biloba* leaves. Industrial Crops Prod. 158, 112980. 10.1016/j.indcrop.2020.112980

[B32] Lukačin R. Matern U. Junghanns K. T. Heskamp M. L. Britsch L. Forkmann G. (2001). Purification and antigenicity of flavone synthase I from irradiated parsley cells. Archives Biochem. Biophysics 393 (1), 177–183. 10.1006/abbi.2001.2491 11516175

[B33] Lukacin R. Wellmann F. Britsch L. Martens S. Matern U. (2003). Flavonol synthase from *Citrus unshiu* is a bifunctional dioxygenase. Phytochemistry 62 (3), 287–292. 10.1016/s0031-9422(02)00567-8 12620339

[B34] Man-Xiu L. (2009). Determination of eight metal elements in Astragalus by microwave digestion-flame atomic absorption spectrometry. J. Anal. Sci. 25, 605–608. 10.1159/000210413

[B35] Mansur A. R. Kim K. J. Kim D.-B. Yoo M. Jang H. W. Kim D.-O. (2020). Matrix solid-phase dispersion extraction method for HPLC determination of flavonoids from buckwheat sprouts. LWT 133, 110121. 10.1016/j.lwt.2020.110121

[B36] Martens S. Forkmann G. Britsch L. Wellmann F. Matern U. Lukačin R. (2003). Divergent evolution of flavonoid 2-oxoglutarate-dependent dioxygenases in parsley 1. FEBS Lett. 544 (1-3), 93–98. 10.1016/S0014-5793(03)00479-4 12782296

[B37] Miean K. H. Mohamed S. (2001). Flavonoid (myricetin, quercetin, kaempferol, luteolin, and apigenin) content of edible tropical plants. J. Agric. Food Chem. 49 (6), 3106–3112. 10.1021/jf000892m 11410016

[B38] Okhti Z. Abdalah M. Basil D. (2021). Phytochemical structure and Biological Effect of *Ginkgo biloba* leaves:A review. Int. J. Pharm. Res. 13, 1138–1143. 10.31838/ijpr/2021.13.02.180

[B39] Otasek D. Morris J. H. Bouças J. Pico A. R. Demchak B. (2019). Cytoscape automation: Empowering workflow-based network analysis. Genome Biol. 20 (1), 185. 10.1186/s13059-019-1758-4 31477170 PMC6717989

[B40] Pei Y. Li R. Fu H. Jing W. Zhou Y. (2008). A new isoflavone glucoside from *Astragalus membranaceus* var. mongholicus. Fitoterapia 78 (7-8), 602–604. 10.1016/j.fitote.2007.04.007 17600636

[B41] Pelletier M. K. Shirley B. W. (1996a). Analysis of flavanone 3-hydroxylase in arabidopsis seedlings (coordinate regulation with chalcone synthase and chalcone isomerase). Plant Physiol. 111 (1), 339–345. 10.1104/pp.111.1.339 8685272 PMC157841

[B42] Pelletier M. K. Shirley B. W. (1996b). Analysis of flavanone 3-hydroxylase in *Arabidopsis* seedlings. Coordinate regulation with chalcone synthase and chalcone isomerase. Plant physiol. 111 (1), 339–345. 10.1104/pp.111.1.339 8685272 PMC157841

[B43] Pertea M. Pertea G. M. Antonescu C. M. Chang T.-C. Mendell J. T. Salzberg S. L. (2015). StringTie enables improved reconstruction of a transcriptome from RNA-seq reads. Nat. Biotechnol. 33 (3), 290–295. 10.1038/nbt.3122 25690850 PMC4643835

[B44] Pu X. Li Z. Tian Y. Gao R. Hao L. Hu Y. (2020). The honeysuckle genome provides insight into the molecular mechanism of carotenoid metabolism underlying dynamic flower coloration. New Phytol. 227, 930–943. 10.1111/nph.16552 32187685 PMC7116227

[B45] Qin W. Xie L. Li Y. Liu H. Fu X. Chen T. (2021). An r2r3-MYB transcription factor positively regulates the glandular secretory trichome initiation in *Artemisia annua* L. Front. plant Sci. 12, 657156. 10.3389/fpls.2021.657156 33897745 PMC8063117

[B46] Santín O. Moncalián G. (2018). Loading of malonyl-CoA onto tandem acyl carrier protein domains of polyunsaturated fatty acid synthases. J. Biol. Chem. 293 (32), 12491–12501. 10.1074/jbc.RA118.002443 29921583 PMC6093242

[B47] Sen S. Chakraborty R. Kalita P. (2020). Rice - not just a staple food: A comprehensive review on its phytochemicals and therapeutic potential. Trends Food Sci. Technol. 97, 1. 10.1016/j.tifs.2020.01.022

[B48] Shan H. Zheng X. Li M. (2019). The effects of Astragalus membranaceus active extracts on autophagy-related diseases. Int. J. Mol. Sci. 20 (8), 1904. 10.3390/ijms20081904 PMC6514605 30999666

[B49] Shen N. Wang T. Gan Q. Liu S. Wang L. Jin B. (2022). Plant flavonoids: Classification, distribution, biosynthesis, and antioxidant activity. Food Chem. 383, 132531. 10.1016/j.foodchem.2022.132531 35413752

[B50] Shirataki Y. Takao M. Yoshida S. Toda S. (1997). Antioxidative components isolated from the roots of *Astragalus membranaceus* Bunge (Astragali Radix). Phytotherapy Res. 11 (8), 603–605. 10.1002/(sici)1099-1573(199712)11:8<603:aid-ptr161>3.0.co;2-u

[B51] Singla R. K. Dubey A. K. Garg A. Sharma R. K. Al-Hiary M. (2019). Natural polyphenols: Chemical classification, definition of classes, subcategories, and structures. J. AOAC Int. 102 (5), 1397–1400. 10.5740/jaoacint.19-0133 31200785

[B52] Song J.-Z. Mo S.-F. Yip Y.-K. Qiao C.-F. Han Q.-B. Xu H.-X. (2007). Development of microwave assisted extraction for the simultaneous determination of isoflavonoids and saponins in Radix Astragali by high performance liquid chromatography. J. Sep. Sci. 30 (6), 819–824. 10.1002/jssc.200600340 17536726

[B53] Song J. Zhou Y.-z. Pang Y.-y. Gao L. Du G.-h. Qin X.-m. (2020). The anti-aging effect of Scutellaria baicalensis Georgi flowers extract by regulating the glutamine-glutamate metabolic pathway in d-galactose induced aging rats. Exp. Gerontol. 134, 110843. 10.1016/j.exger.2020.110843 32045633

[B54] Su H.-f. Shaker S. Kuang Y. Zhang M. Ye M. Qiao X. (2021). Phytochemistry and cardiovascular protective effects of huang-qi (Astragali Radix). Med. Res. Rev. 41 (4), 1999–2038. 10.1002/med.21785 33464616

[B55] Subarnas A. Oshima Y. Hikino H. (1991). Isoflavans and a pterocarpan from *Astragalus mongholicus* . Phytochemistry 30 (8), 2777–2780. 10.1016/0031-9422(91)85143-n

[B56] The UniProt C. (2021). UniProt: The universal protein knowledgebase in 2021. Nucleic Acids Res. 49 (D1), D480–D489. 10.1093/nar/gkaa1100 33237286 PMC7778908

[B57] Tohge T. de Souza L. P. Fernie A. R. (2017). Current understanding of the pathways of flavonoid biosynthesis in model and crop plants. J. Exp. Bot. 68 (15), 4013–4028. 10.1093/jxb/erx177 28922752

[B58] Torres N. Martínez-Lüscher J. Porte E. G. Kurtural S. K. (2020). Optimal ranges and thresholds of grape berry solar radiation for flavonoid biosynthesis in *warm climates* . Front. Plant Sci. 11, 931. 10.3389/fpls.2020.00931 32714350 PMC7344324

[B59] Turnbull J. J. Nakajima J.-i. Welford R. W. D. Yamazaki M. Saito K. Schofield C. J. (2004). Mechanistic studies on three 2-oxoglutarate-dependent oxygenases of flavonoid biosynthesis: Anthocyanidin synthase, flavonol synthase, and flavanone 3beta-hydroxylase. J. Biol. Chem. 279 (2), 1206–1216. 10.1074/jbc.M309228200 14570878

[B60] Vetten N. D. Horst J. T. Schaik H. (1999). A cytochrome b5 is required for full activity of flavonoid 3′,5′-hydroxylase, a cytochrome P450 involved in the formation of blue flower colors. Proc. Natl. Acad. Sci. U. S. A. 96 (2), 778–783. 9892710 10.1073/pnas.96.2.778 PMC15213

[B61] Wan Q. Bai T. Liu M. Liu Y. Xie Y. Zhang T. (2022). Comparative analysis of the chalcone-flavanone isomerase genes in six *citrus* species and their expression analysis in sweet orange (*Citrus sinensis*). Front. Genet. 13, 848141. 10.3389/fgene.2022.848141 35495138 PMC9039136

[B62] Wang B. Chen H. Ma H. Zhang H. Lei W. Wu W. (2016). Complete plastid genome of *Astragalus membranaceus* (Fisch.) Bunge var. membranaceus. Mitochondrial DNA. Part B, Resour. 1 (1), 517–519. 10.1080/23802359.2016.1197057 PMC7800798 33473540

[B63] Wang C. Dong Y. Zhu L. Wang L. Yan L. Wang M. (2020a). Comparative transcriptome analysis of two contrasting wolfberry genotypes during fruit development and ripening and characterization of the LrMYB1 transcription factor that regulates flavonoid biosynthesis. BMC genomics 21 (1), 295. 10.1186/s12864-020-6663-4 32272876 PMC7147035

[B64] Wang L. Cui J. Jin B. Zhao J. Xu H. Lu Z. (2020b). Multifeature analyses of vascular cambial cells reveal longevity mechanisms in old *Ginkgo biloba* trees. Proc. Natl. Acad. Sci. U. S. A. 117 (4), 2201–2210. 10.1073/pnas.1916548117 31932448 PMC6995005

[B65] Wang N. Xu H. Jiang S. Zhang Z. Lu N. Qiu H. (2017a). MYB12 and MYB22 play essential roles in proanthocyanidin and flavonol synthesis in red-fleshed apple (*Malus sieversii* f. niedzwetzkyana). Plant J. 90 (2), 276–292. 10.1111/tpj.13487 28107780

[B66] Wang Q, H. Han N, R. C. K. T. Dai N, Y. T. Wang X. L. Ao W. L. J. (2014). Anti-inflammatory effects and structure elucidation of two new compounds from *Astragalus membranaceus* (Fisch) Bge. var. mongholicus (Bge) Hsiao. J. Mol. Struct. 1074 (285), 284–288. 10.1016/j.molstruc.2014.06.020

[B67] Wang S. Yang C. Tu H. Zhou J. Liu X. Cheng Y. (2017b). Characterization and metabolic diversity of flavonoids in *citrus* species. Sci. Rep. 7 (1), 10549. 10.1038/s41598-017-10970-2 28874745 PMC5585201

[B68] Wang X. Hu H. Wu Z. Fan H. Wang G. Chai T. (2021). Tissue-specific transcriptome analyses reveal candidate genes for stilbene, flavonoid and anthraquinone biosynthesis in the medicinal plant Polygonum cuspidatum. BMC genomics 22 (1), 353. 10.1186/s12864-021-07658-3 34000984 PMC8127498

[B69] Wang Z. Gerstein M. Snyder M. (2009). RNA-seq: A revolutionary tool for transcriptomics. Nat. Rev. Genet. 10 (1), 57–63. 10.1038/nrg2484 19015660 PMC2949280

[B70] Xi Z. Xiao H. B. Xue X. Y. Sun Y. G. Liang X. M. (2015). Simultaneous characterization of isoflavonoids and astragalosides in two *Astragalus* species by high-performance liquid chromatography coupled with atmospheric pressure chemical ionization tandem mass spectrometry. J. Sep. ence 30 (13), 2059–2069. 10.1002/jssc.200700014 17657828

[B71] Xue X. Zhao A. Wang Y. Ren H. Du J. Li D. (2021). Composition and content of phenolic acids and flavonoids among the different varieties, development stages, and tissues of Chinese Jujube (*Ziziphus jujuba* Mill.). PloS one 16 (10), e0254058. 10.1371/journal.pone.0254058 34648512 PMC8516285

[B72] Yan N. Du Y. Liu X. Chu M. Shi J. Zhang H. (2019). A comparative UHPLC-QqQ-MS-based metabolomics approach for evaluating Chinese and North American wild rice. Food Chem. 275, 618–627. 10.1016/j.foodchem.2018.09.153 30724241

[B73] Yuan L. Pan K. Li Y. Yi B. Gao B. (2021). Comparative transcriptome analysis of Alpinia oxyphylla Miq. reveals tissue-specific expression of flavonoid biosynthesis genes. BMC genomic data 22 (1), 19. 10.1186/s12863-021-00973-4 34090339 PMC8180045

[B74] Yuan T. Fujioka S. Takatsuto S. Matsumoto S. Gou X. He K. (2007). BEN1, a gene encoding a dihydroflavonol 4-reductase (DFR)-like protein, regulates the levels of brassinosteroids in *Arabidopsis thaliana* . Plant J. 51 (2), 220–233. 10.1111/j.1365-313X.2007.03129.x 17521414

[B75] Yuan Y. Zhang J. Liu X. Meng M. Wang J. Lin J. (2020). Tissue-specific transcriptome for Dendrobium officinale reveals genes involved in flavonoid biosynthesis. Genomics 112 (2), 1781–1794. 10.1016/j.ygeno.2019.10.010 31678153

[B76] Zhang Y. Gu L. Xia Q. Tian L. Qi J. Cao M. (2020). Radix Astragali and Radix angelicae sinensis in the treatment of idiopathic pulmonary fibrosis: A systematic review and meta-analysis. Front. Pharmacol. 11, 415. 10.3389/fphar.2020.00415 32425767 PMC7203419

[B77] Zhao C. Liu X. Gong Q. Cao J. Shen W. Yin X. (2021). Three AP2/ERF family members modulate flavonoid synthesis by regulating type IV chalcone isomerase in citrus. Plant Biotechnol. J. 19 (4), 671–688. 10.1111/pbi.13494 33089636 PMC8051604

[B78] Zheng K. Choi R. Cheung A. Guo A. Bi C. Zhu K. Y. (2011). Flavonoids from Radix Astragali induce the expression of erythropoietin in cultured cells: A signaling mediated via the accumulation of hypoxia-inducible factor-1α. J. Agric. Food Chem. 59 (5), 1697–1704. 10.1021/jf104018u 21309574

