# Supplementary material for: Transcriptional regulation mechanism of flavonoids biosynthesis gene during fruit development in astragalus membranaceus
Source: Front Genet. 2022 Sep 6;13:972990. doi: 10.3389/fgene.2022.972990 (PMC9485568; doi:10.3389/fgene.2022.972990)
Supplement: Supplementary file 5 [file DataSheet1.docx]

Supplementary Material

# 1 Supplementary Figures and Tables

## 1.1 Supplementary Figures


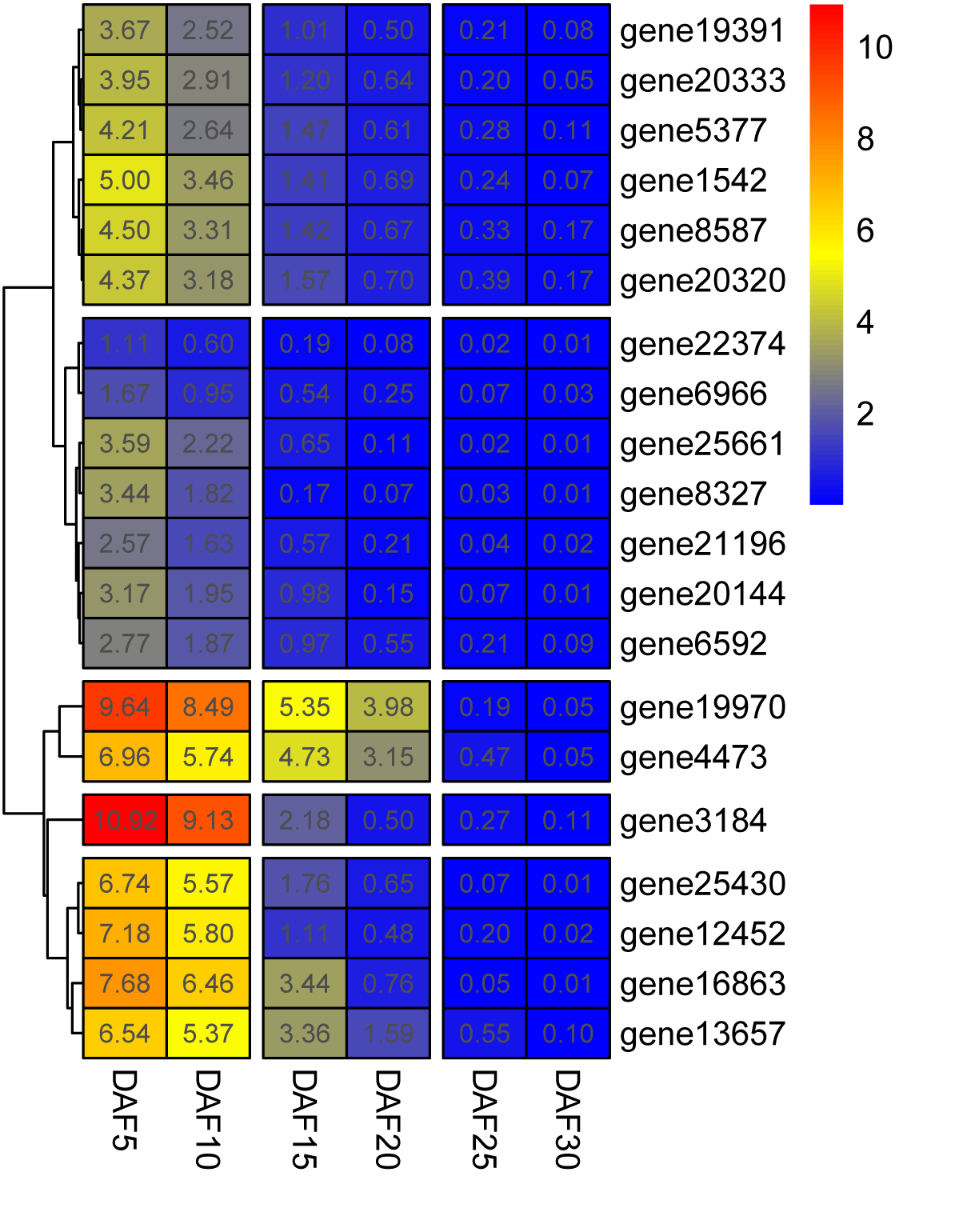


**Figure S1.** Heat map for transcript abundance of the shared genes in among the up-regulated DEGs and the quantitative values were processed with log_2_ (RPKM+1).


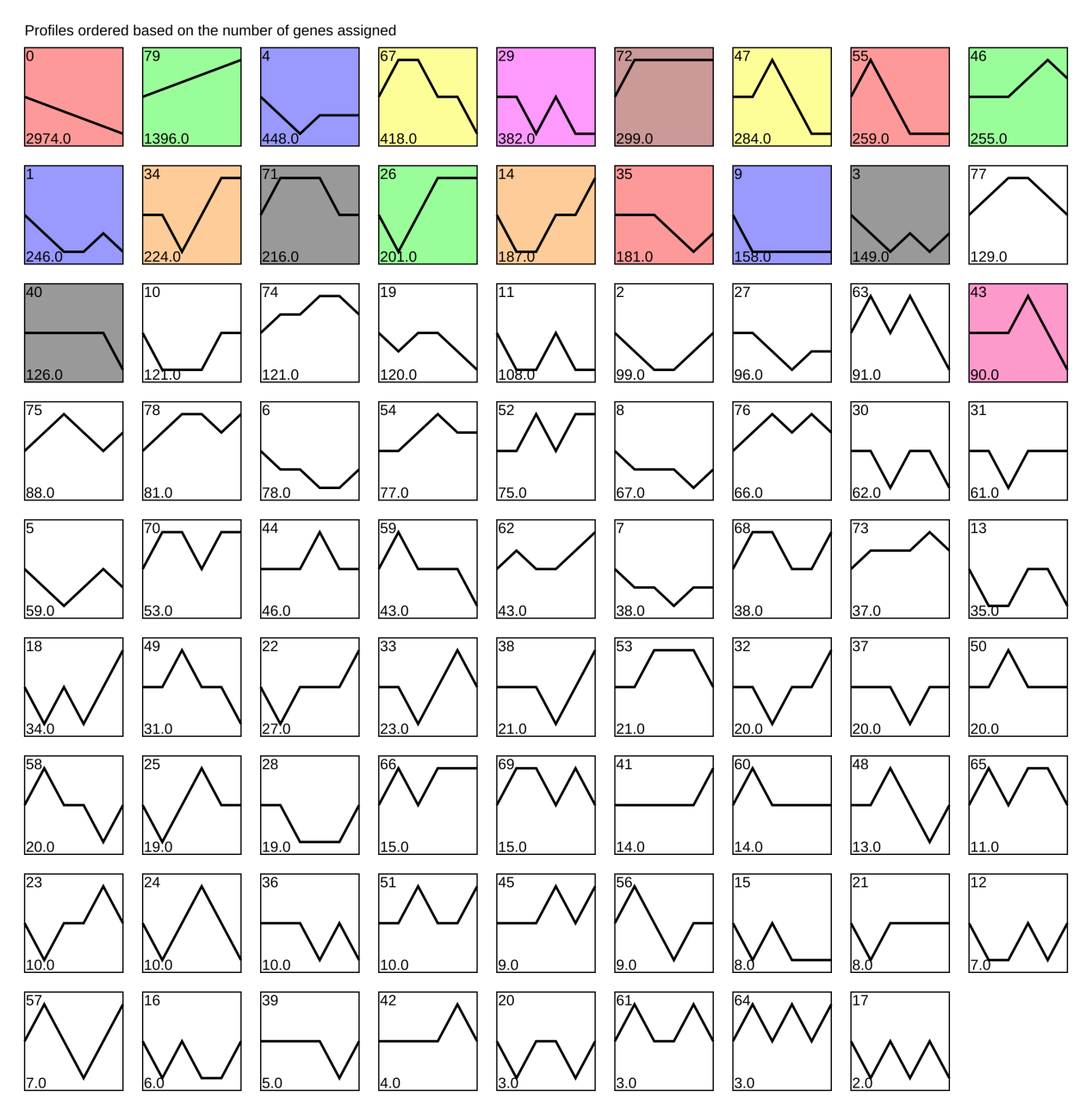


**Figure S2.** Profiles ordered based on the number of genes assigned.


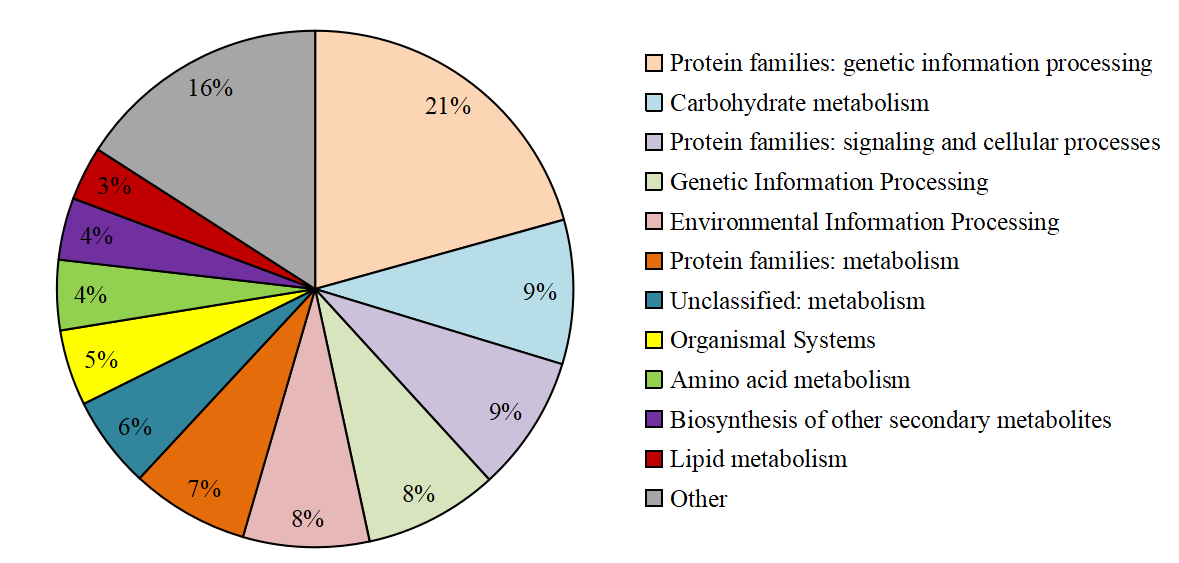


**Figure S3.** Gene enrichment with p[athway](https://www.kegg.jp/kegg-bin/find_pathway_object?query_pathwayfile=523d4399a752faa00dfb227e03dbeab04f223238/user_ko.txt) of KEGG in profile 0.


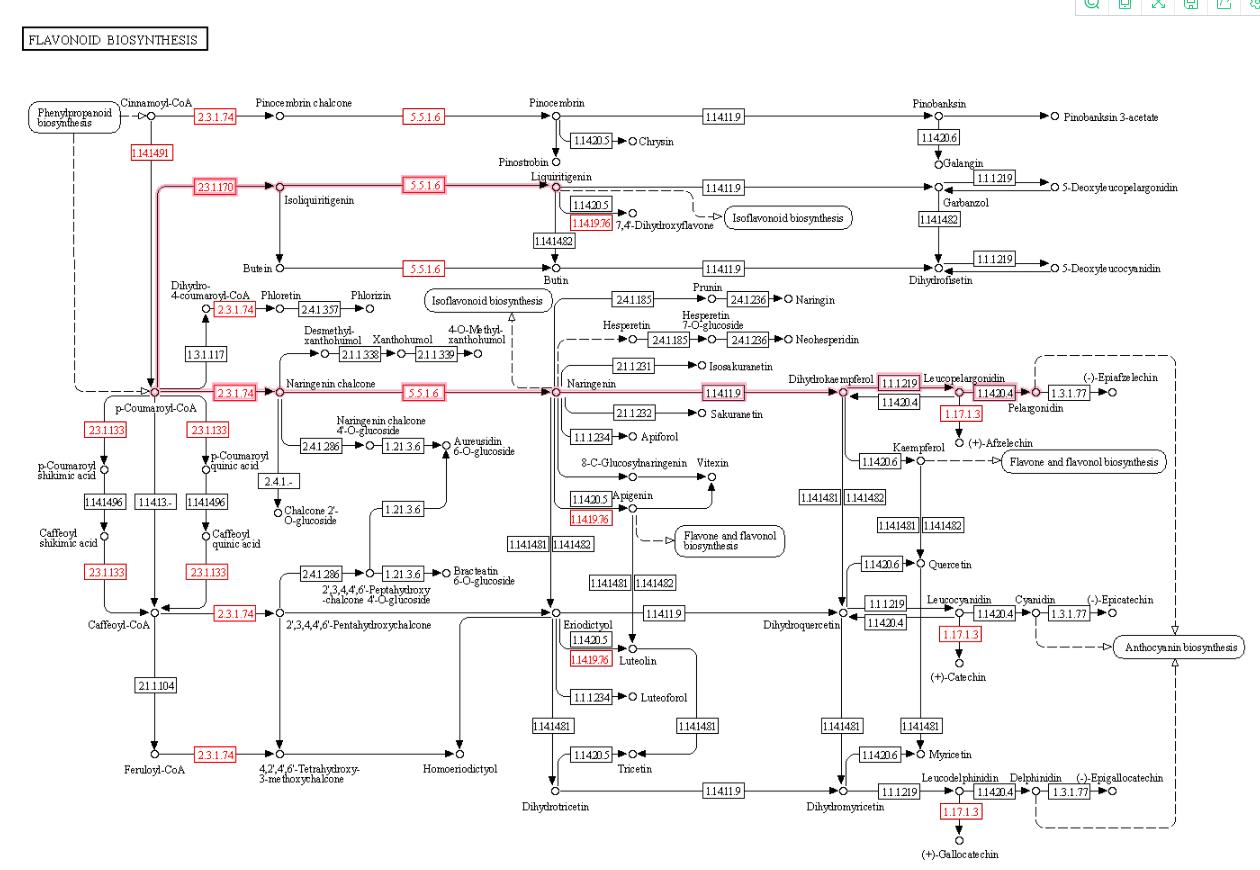


**Figure S4.** Flavonoid biosynthesis pathway and flavonoid related genes at six fruit developmental stages.

## 1.2 Supplementary Tables

**Table S1.** Gene Ontology terms for genes in the profile 0.

**Table S2.** Gene Ontology terms for genes in the profile 79.

**Table S3.** Protein-protein interaction network of flavonoid related proteins in *A. memeranaceus* according to *A. thaliana* paralogous proteins.
